# Auditory Processing after Early Left Hemisphere Injury: A Case Report

**DOI:** 10.3389/fneur.2017.00226

**Published:** 2017-05-24

**Authors:** Cristina Ferraz Borges Murphy, Georgios Stavrinos, Kling Chong, Tony Sirimanna, Doris-Eva Bamiou

**Affiliations:** ^1^The Ear Institute, University College London, London, UK; ^2^Radiology Department, Great Ormond Street Hospital, London, UK; ^3^Audiological Medicine Department, Great Ormond Street Hospital, London, UK

**Keywords:** acquired auditory processing disorder, early brain injury, language reorganization, left hemisphere lesion, auditory perception

## Abstract

Few studies have addressed the long-term outcomes of early brain injury, especially after hemorrhagic stroke. This is the first study to report a case of acquired auditory processing disorder in a 10-year-old child who had a severe left hemorrhagic cerebral infarction at 13 months of age, compromising nearly all of the left temporal lobe. This case, therefore, is an excellent and rare opportunity to investigate the presence of neural plasticity of central auditory system in a developing brain followed severe brain damage. After assuring normal functioning of the peripheral auditory system, a series of behavioral auditory processing tests was applied in dichotic and monaural listening conditions and with verbal and non-verbal stimuli. For all verbal dichotic tasks (dichotic digits, competing words, and sentences tests), good performance on the left ear, especially for Dichotic digits test (100%), and zero performance on the right ear were observed. For monaural low-redundancy tests, the patient also exhibited good performance for auditory figure-ground and time-compressed sentences tests in the left ear. In the right ear, a very poor performance was observed, but slightly better than the same in Dichotic tasks. Impaired performance was also observed in the LiSN test in terms of spatial advantage and, for the Pitch Pattern Sequence test, the only non-verbal test applied, the patient had performance within the normal range in both ears. These results are interpreted taking into consideration the anatomical location of stroke lesion and also the influence of hemispheric specialization for language on auditory processing performance.

## Introduction

The patient was a 10-year-old male, who had a left hemorrhagic cerebral infarction secondary to arteriovenous malformation (AVM) at 13 months of age. Because of his significant difficulty related to auditory perception, he was referred to the Audiological Medicine department at Great Ormond Street Hospital for Children to undergo hearing assessments, including auditory processing evaluation at the age of 10 years.

**Figure 1:**
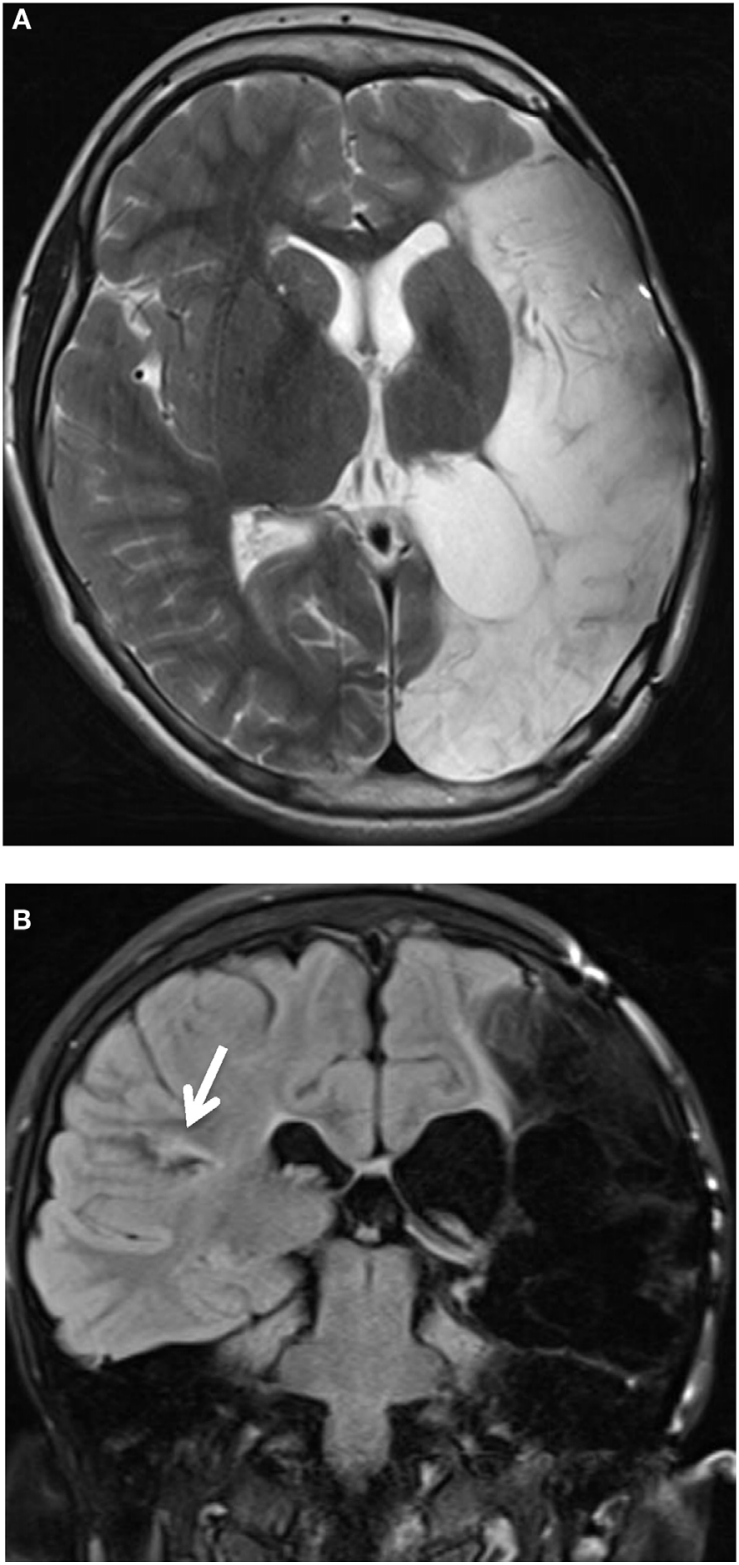
**Axial T2-weighted image**. **(A)** Presence of left craniectomy. Extensive mature injury mostly involving the left middle and posterior cerebral artery territories. Nearly all of the left temporal lobe was involved (not shown). **(B)** Coronal T2-weighted FLAIR image. Less extensive mature regions of cortical injury, best seen in the right posterior sylvian cortex (white arrow) and right parietal cortex. Symmetrical mature injury of the cerebellar hemispheres was also noted (not shown).

His latest MRI (at age 11) showed, apart from the left craniectomy, an extensive mature injury in the left hemisphere mostly involving the left middle and posterior cerebral artery territories and nearly the entire left temporal lobe. There were less extensive mature regions of cortical injury in the right hemisphere, best seen in the right posterior sylvian cortex and right parietal cortex. Symmetrical mature injury of the cerebellar hemispheres was also noted (Figure 1).

Due to the extensive brain hemorrhage, currently, the patient still presents a variety of neurological sequelae and has been followed up by a multidisciplinary clinical team composed by neuropediatrician, speech therapist, physiotherapist, occupational therapist, and psychologist. His latest assessment report detected cerebral visual impairment, which consists of any type of visual impairment related to brain damage or dysfunction (1), peripheral visual field loss, and minimal selective movement in his right hand, due to the cerebral palsy.

On history taking regarding his auditory skills, his mother reported difficulties with hearing, especially in terms of sound location skills, understanding, and responding to spoken language, especially in the presence of background noise. His difficulties include following oral instructions, discriminating speech sounds, giving eventually inconsistent responses to auditory information. To facilitate conversation, people need to repeat and slow down the rate of speech. Cognitive complaints were also reported including short-term memory (visual and auditory) and attention (visual and auditory fatigue), as well as delayed speech language. Despite that, the patient has an acceptable performance at school, e.g., reading and writing satisfactorily, through the adoption of special education needs strategies, such as touch typing to write and talking technology to read.

First, a basic audiological evaluation was conducted to assess the function and integrity of the peripheral auditory system. The following tests were applied: pure one audiometry (GSI 61; Grason Stadler), tympanometry and stapedial reflexes with ipsi- and contralateral stimulation (GSI 33 Middle Ear Analyser, Grason Stadler), transient evoked otoacoustic emissions (Otodynamics Echoport ILO292). This basic audiological evaluation revealed normal pure tone thresholds in both ears, normal middle ear pressure and compliance record from both ears, normal reflexes with both ipsi- and contralateral stimulation for both ears, and normal cochlear outer hair cell function bilaterally. These results confirmed no impairments in the peripheral auditory system, including the outer, middle and inner ear, auditory nerve, and low brainstem.

An auditory processing evaluation was also carried out to assess the auditory functions of the brain in terms of speech and non-speech discrimination. A variety of tests were included in order to assess the main auditory processes such as auditory discrimination, auditory temporal, and pattern processing, dichotic listening, auditory performance in competing acoustic signals, and with degraded speech (2). All tests except the LiSN-S were conducted via a CD player routed via the GSI 61 audiometer. The following tests were applied: speech audiometry (word discrimination test), the monaural low-redundancy tests auditory figure-ground + 8 dB/SCAN-3:C (3), filtered words/SCAN-3:C (3) and time-compressed sentences (60% compression)/SCAN-3:C (3), the dichotic tests’ Dichotic digits (4, 5), competing words/SCAN-3:C (3) and competing sentences/SCAN-3:C (3), the auditory temporal test Pitch Pattern Sequence with verbal response (5, 6), and the auditory spatial test Listening Spatialized Noise–Sentences Test/LiSN-S (conducted via a calibrated laptop and Senheiser headphones) (7, 8). The monaural low-redundancy tests assess the abilities of auditory closure and speech perception in noise. The speech signal is degraded (filtered speech and time-compressed sentences tests) or embedded in competing signal (auditory figure-ground test), reducing its natural redundancy. Dichotic tests assessed binaural integration skills, which is the ability to process different stimuli presented to each ear at the same time. The pitch pattern sequence test assesses the ability to discriminate and recognize the sequence of non-verbal stimuli that differs in terms of frequency. LiSN-S is an auditory spatial processing test that assesses the ability to use the spatial cues to differentiate a target talker from distracting speech sounds.

The scores obtained and the interpretation of results is demonstrated in Table 1. The scores were transformed in percentiles and analyzed according to the age norms specific for each test.

**Table 1:** **Auditory processing tests performance**.

Tasks	Patient performance		Interpretation of results	
	LE	RE	LE	RE
**Speech audiometry**				
Word discrimination	40 dB/60%	50 dB/20%	Poor discrimination	
**Monaural low-redundancy**				
Auditory figure-ground	85%	30%	5th (borderline)	0.1 (impaired)
Filtered words	25%	5%	0.1 (impaired)	0.1 (impaired)
Time-compressed sentences	86%	3%	16th (normal)	0.1 (impaired)
**Dichotic tasks**				
Dichotic digits	100%	0%	98th (normal)	0.1 (impaired)
Competing words	60%	0%	25th (normal)	0.1 (impaired)
Competing sentences	60%	0%	2nd (borderline)	0.1 (impaired)
**Pattern tasks**				
Pitch pattern test	75%	60%	19th (normal)	5th (borderline)
**Auditory spatial test**				
LiSN				
Talker advantage	2.5 dB		Normal	
Spatial advantage	3.6 dB		Impaired	

A severely impaired performance was observed in the right ear, in all the tests, except in the pitch pattern test, which was the only non-verbal test applied. In this specific test, the performance was nearly within the normal range for his age (5). In the left ear, the performance was within the normal range to mildly impaired in most of the tests, except in the filtered speech test, in which performance was very poor. The LiSN test showed impairment related to the spatial advantage, indicating a deficit related to spatial stream segregation. Overall, these results indicated a severe acquired auditory processing disorder (9), especially related to speech perception.

**Figure 2:**
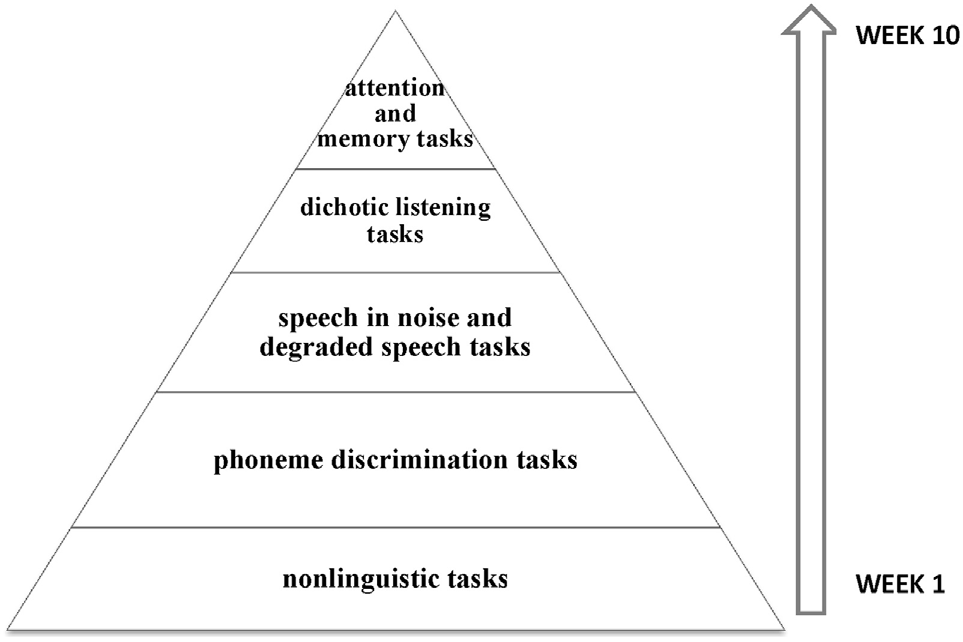
**Schematic representation of auditory trained skills**. The training started with less complex stimuli and tasks, such as the tasks involving frequency discrimination. Gradually, speech stimuli and also complex tasks involving speech with background noise were introduced, especially focused on the right ear. In the last weeks, top-down tasks involving working memory and sustained attention were included.

An intensive home-based computer auditory training was recommended for 10 weeks, comprising non-linguistic and linguistic tasks, speech discrimination tasks with and without background noise, and top-down tasks involving attention and memory (Figure 2).

During the training, the patient showed slight but progressive improvements on the trained tasks performance; therefore, we suggested continuing practicing the auditory skills even after the intensive 10 weeks of training and the performance would be periodically monitored. The patient has also used FM system at school (10) in order to better understand his teacher.

## Background and Discussion

Stroke is one of the main causes of death in childhood (11), with an incidence of 1.2–13 cases per 1,000,000 children under 18 years old (12). More specifically, hemorrhagic strokes due to AVM, such as the case reported, are the most common cause of hemorrhagic stroke after infancy (13).

Few studies have addressed the long-term outcomes of early brain injury, especially after hemorrhagic stroke (14). In cases of left hemisphere lesions, most research has focused on the impact of the brain injury on language development, especially in terms of brain language reorganization (15–20), but without taking into consideration the auditory perception perspective. According to the British Society of Audiology (9), acquired auditory processing disorder, as reported here, is associated with a known postnatal event, such as neurological trauma or infection, which could probably explain the auditory processing disorder.

This is the first study to report a case of acquired auditory processing disorder in a 10-year-old child who had a severe left hemorrhagic cerebral infarction at 13 months of age, compromising nearly all of the left temporal lobe. The case is special because no study has reported the long-term outcomes of severe early brain injury in relation to auditory processing skills. Therefore, it is an excellent and rare opportunity to investigate the presence of neural plasticity of central auditory system in a developing brain followed brain damage.

In the following discussion, the results have been interpreted through the interaction between auditory processing tests performance, language, and clinical presentation.

### Right × Left Ear Performance on Dichotic Tests

The good performance on the left ear versus zero performance on the right ear in all verbal dichotic tests (Dichotic Digits, competing words, and sentences tests) might indicate reorganization and development of the language in the right hemisphere as a result of the strong plasticity that generally follows early brain injury (15, 16, 18). In fact, research has demonstrated that, in perinatal brain injuries, due to the highest brain plasticity, the damaged hemisphere might continue developing control over language, maintaining the genetic predisposition. On the other hand, in at term or early development brain injuries, as the present case, because the plasticity is relatively reduced, the non-damaged hemisphere might dominate over the damage one and assume the control of language (21). This transfer of verbal functions to the right hemisphere after early left hemisphere lesions has been demonstrated in several studies by different methods (16–18, 20). One of them is the dichotic listening paradigm, as applied in the present study (20, 22, 23). Under dichotic conditions, when the individual is required to report different verbal stimuli presented simultaneously to each ear, the stimuli are predominantly processed via contralateral pathways due to suppression of the ipsilateral pathways (24–26). Therefore, in most cases, due to the left hemisphere specialization for language, a better right ear performance is observed, the so-called “right ear advantage” phenomenon (25, 27). In this study, because the advantage was observed in the left ear (“left ear advantage”), we might hypothesize that the right hemisphere assumed dominant language functions, as previous research has shown (20, 22, 23). This hypothesis may also be reinforced by the presence of right hemiplegia, which is an indicator of severe left cerebral dysfunction and it is highly associated with language organization exclusively to the right hemisphere (28). Despite that, the possibility of right language organization prestroke, although rare (29), should not be ruled out, even taking into account that the patient has no close left-handed relatives.

Besides the left ear advantage, demonstrated in all the three dichotic tests, the dichotic digit performance on the left ear was considerably better than performance in the competing words which was, in turn, better than performance in competing sentences in the same ear. These results might be associated with linguistic demands involved in each task; dichotic digits is a closed set speech perception task, while competing words and sentences tests are relatively more challenging, demanding, for instance, greater vocabulary (30). Despite this, the patient had a performance only slightly below the normal range in the left ear competing sentences, which might indicate the plastic capacity of the right hemisphere to mediate satisfactory language capabilities, with only some subtle deficits (31), as presented by the patient.

### Dichotic × Monaural Tests – Ipsi × Contralateral Pathways

**Figure 3:**
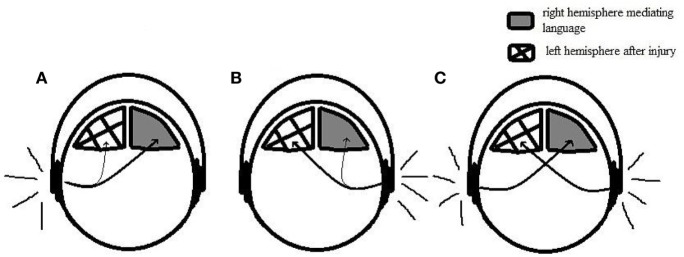
**Illustration representing monaural and dichotic conditions**. **(A)** Monaural condition with speech stimuli presented on the left ear. Contralateral pathways transport the speech stimuli to the right hemisphere, where it is successfully processed. **(B)** Monaural condition with speech stimuli presented on the right ear. Ipsilateral pathways transport the stimuli to the right hemisphere; however, it is not properly conducted due to the weakness of the ipsilateral tract. **(C)** Dichotic condition. Contralateral pathways transport the speech stimuli to the opposite hemisphere, leading to a good performance on the left ear and poor performance on the right ear.

While the performance on the right ear was null in the dichotic tests, in the monaural low-redundancy tests, the performance was slightly better, but, still very poor. This relatively “better” performance might be related to the involvement of the ipsilateral pathways to transport the verbal stimuli on this condition (32). Figure 3 illustrates the differences in terms of physiological mechanisms when the stimuli are presented dichotically and monaurally taking into account the left brain injury and the hypothesis of reorganization of language function on the right hemisphere.

While in the dichotic conditions, the ipsilateral pathways are suppressed by contralateral pathways leading to the null performance on those tests, in the monaural speech tests, such as the speech audiometry and all the monaural low-redundancy tests, there is the involvement of both pathways. The ipsilateral pathway, therefore, might transport the stimuli from the right ear to the right hemisphere, enabling the auditory processing of speech stimuli through the right hemisphere. Despite that, the performance on the right ear was still very poor. Taking into account the very good performance on the left ear, which demonstrates the good capacity of the right hemisphere to handle the task, we can hypothesize that the poor performance in the right ear might be related to the weakness of the ipsilateral tract, which has much less connection in the central nervous systems than the contralateral pathways (24) and might not convey the speech stimuli effectively.

While the performance in the auditory figure-ground and time-compressed sentences test was poor only in the right ear, in the filtered words, the performance was impaired in both ears. Although all these tests involve degraded speech, different speech domains are manipulated in each one of them (temporal degradation on time-compressed speech test and spectral degradation on speech in noise and filtered speech), which is likely to lead to differences in terms of the sensitivity to central auditory nervous system lesions (33). Previous research involving diffusing tensor imaging (DTI), demonstrated, for instance, positive correlations between the DTI parameter and the filtered words test performance in the corpus callosum. The same positive correlation was found in the prefrontal areas for the speech-in-noise and time-compressed speech test (60% compression) (34). In the case reported, according to the MRI, the right prefrontal cortex was preserved and the corpus callosum showed abnormal signal in the splenium likely due to the secondary Wallerian degeneration. These MRI observations might be, therefore, in line with the previous findings; the intact right prefrontal cortex might explain the good performance on both auditory figure-ground and time-compressed sentences tests in the left ear while the left ear impaired performance in the filtered words test might be associated with the corpus callosum injury.

### Verbal × Non-Verbal Tests

In the only non-verbal test applied (Pitch Pattern Sequence test), the patient had performance within the normal range in both ears. In fact, it was the only auditory processing test applied with normal performance in both ears. This result differs from the previous study that has demonstrated poor performance of individuals with cerebral lesions on this same test, regardless the hemisphere or site of lesion (35). Through this previous study, it has been assumed that this test has a high sensitivity to cerebral lesions given that it involves several areas of the brain, in both hemispheres such as the primary auditory cortex (transverse gyri of Heschl) in both hemispheres, auditory association areas, and language areas of the temporoparietal area, in case of verbal response (35). The activation of many cerebral areas are linked with the several neural processes that this task demands; the recognition of acoustic contours and pattern, for instance, has been associated, predominantly, to the right hemisphere, while the linguistic labeling (when the test requires verbal response) has been associated with the language areas in the left hemisphere (35). The difference between the current and previous findings is likely due to the fact that the previous study was conducted on acquired auditory processing disorder during adulthood as opposed on early stages of development in the present case study.

In this case, the successful performance on this test might indicate, the intact ability of the right hemisphere to successfully recognize acoustic contours and pattern, which was already expected to occur in the right hemisphere (35). In addition, it might also demonstrate, at least, a reasonable auditory short-term memory to successfully store and retrieve the stimuli (36), and finally, the capacity of the right hemisphere to linguistically label the stimuli, due to the likely transfer of verbal functions to the right hemisphere. In short, it might demonstrate the ability of the right hemisphere to process auditory sensory, cognitive, and linguistic information related to the stimuli, such as labeling it.

The patient also had impaired performance in the LiSN test in terms of spatial advantage, suggesting, *a priori*, a deficit involving auditory stream segregation skill (7, 8). However, this task also involves binaural integration skill, which would also lead to the hypothesis that associates this performance to the impairment of the right ear in tests involving auditory performance in competing acoustic signals. Few studies have investigated the neural correlates of spatial stream segregation tasks in general (37, 38). A recent study conducted in rats demonstrated that not only the auditory cortex is associated with spatial stream segregation but also the ascending auditory pathway, through the gradual sharpening of spatial sensitivity on the brainstem and thalamus and the forward suppression between thalamic and cortical levels (37). Due to the involvement of several levels of the ascending auditory pathway, the interpretation of the present results is not straightforward. Further studies involving neurological population and fMRI are necessary to better understand the extent to which brainstem and auditory cortex are related to this specific skill. Contrary to the spatial advantage, talker advantage performance was normal, which might indicate, according to the previous study (39), preservation of vocal tract parameter encoding in the right hemisphere, consistent with the present imaging results.

## Concluding Remarks

Overall, the results demonstrated good performance on the left ear and zero or slightly better performance on the right ear for all verbal tests applied, which might reinforce the differences in terms of physiological mechanisms when dichotic and monoaural listening condition are applied. In addition, the hypothesis of language reorganization in the right hemisphere was also debated. The performance within the normal range, in both ears, in the frequency pattern test, might demonstrate the plastic capacity of the right hemisphere to operate several neural processes involving sensory, cognitive, and linguistic aspects, after an early life brain injury.

This is a very instructive case to understand the influence of early left hemisphere injury on auditory processing development. It also shows the role of hemispheric specialization for language on auditory processing performance and the importance of considering different paradigms such as verbal and non-verbal auditory tests, dichotic and monaural tests, for better interpretation of the processes that underlie each auditory processing task. Further fMRI studies on children with severe early left hemisphere injury are required to better investigate the neural correlates of different auditory processing skills.

## Ethics Statement

The patient’s mother obtained verbal assent from the child and provided written consent for publication of this case report.

## Author Contributions

CM: conception, draft and revision of the work, analysis, acquisition, and interpretation of data. GS: contribution to the conception and revision of the work, acquisition, and interpretation of data. KC: contribution to the conception, revision of the work, and interpretation of data. TS: contribution to the conception and revision of the work. D-EB: conception, draft, and revision of the work and interpretation of data. CM, GS, KC, TS, and D-EB: final approval of the version to be published.

## Conflict of Interest Statement

The authors declare that the research was conducted in the absence of any commercial or financial relationships that could be construed as a potential conflict of interest.
